# Chronic hepatitis C treatment in HIV co‐infection in Portugal: Results from a cohort OF 2133 patients presented by GEPCOI (Portuguese Coinfection Study Group)

**DOI:** 10.1111/jvh.13281

**Published:** 2020-03-11

**Authors:** Ana Cláudia Miranda, Josefina Mendez, Rosário Serrão, Francisco Vale, Maria José Manata, Sara Pinto, André Gomes, Cristina Valente, Patrícia Pacheco, Rosário Pazos, Rui Pereira, Ana Martins, Isabel Germano, Sónia Rocha, Ana Paula Reis, Rui Sarmento‐Castro

**Affiliations:** ^1^ Serviço de Infecciologia e Medicina Tropical Centro Hospitalar de Lisboa Ocidental Hospital de Egas Moniz Lisboa Portugal; ^2^ Serviço de Doenças Infecciosas Centro Hospitalar do Porto Porto Portugal; ^3^ Serviço de Doenças Infecciosas Centro Hospitalar de São João Porto Portugal; ^4^ Serviço de Doenças Infecciosas Centro Hospitalar de Setúbal Setúbal Portugal; ^5^ Serviço de Doenças Infecciosas Centro Hospitalar Universitário de Lisboa Central Hospital de Curry Cabral Lisboa Portugal; ^6^ Serviço de Doenças Infecciosas Centro Hospitalar de Gaia/Espinho Gaia/Espinho Portugal; ^7^ Serviço de Infecciologia Hospital Garcia de Orta, EPE Almada Portugal; ^8^ Serviço de Doenças Infecciosas Centro Hospitalar e Universitário de Coimbra Coimbra Portugal; ^9^ Serviço de Doenças Infecciosas Hospital Fernando da Fonseca Amadora Portugal; ^10^ Serviço de Medicina Centro Hospitalar Universitário do Algarve, Hospital de Portimão Portimão Portugal; ^11^ Serviço de Doenças Infecciosas Centro Hospitalar Universitário do Algarve Hospital de Faro Faro Portugal; ^12^ Serviço de Doenças Infecciosas Centro Hospitalar do Baixo Vouga Aveiro Portugal; ^13^ Serviço de Medicina 1.4 Centro Hospitalar Universitário de Lisboa Central, Hospital de São José Lisboa Portugal; ^14^ Serviço de Doenças Infecciosas Unidade Local Saúde Alto Minho Viana do Castelo Portugal; ^15^ Serviço de Doenças Infecciosas Hospital dos Marmeleiros Funchal Portugal

**Keywords:** DAA treatment, GEPCOI, HCV, HIV co‐infection, Portugal

## Abstract

Direct‐acting antiviral drugs (DAAs) have recently changed the paradigm of hepatitis C therapy, significantly improving treatment response rates, patient life expectancy and quality of life. In Portugal, sofosbuvir (SOF) and SOF/ledipasvir (SOF/LDV) were fully reimbursed by the National Health System since early 2015 and generalized use of interferon‐free DAA based regimens became current practice. During 2016, the remaining DAAs were sequentially added and covered by the same health access policy. The Portuguese Study Group of Hepatitis and HIV Co‐infection (GEPCOI) collected data from 15 clinical centres in Portugal, pertaining to the HCV treatment experience with DAA regimens. A cohort of 2133 patients was analysed, representing one of the largest DAA treated HCV/HIV co‐infected individuals. The global sustained virologic response (SVR) achieved was 95% in this real‐life cohort setting. Linear regression analysis showed significant differences in treatment response rates when using SOF plus ribavirin (RBV) combination in genotype 2 or 3 infected individuals (*P* < .002) and in those with liver cirrhosis (*P* < .002). These findings corroborate that early treatment is mandatory in HIV/HCV co‐infected patients, as response rates may be negatively influenced by higher fibrosis stages and suboptimal DAA regimens. The current national Portuguese health policy should continue to promote wider treatment access and individualized therapy strategies, aiming at the elimination of HCV infection in this high‐risk co‐infected population.

**Abbreviations**
DAAs: direct‐acting antiviral drugs
FS: fibroscan score
GEPCOI: Portuguese Study Group of Hepatitis and HIV Co‐infection
HCV: Hepatitis C virus
HIV: human immunodeficiency virus
LDV: ledipasvir
ORs: Odds ratios
RBV: ribavirin
SOF: sofosbuvir
SVR: sustained virologic response

## 1 INTRODUCTION

Direct‐acting antiviral drugs (DAAs) have recently changed the paradigm of chronic hepatitis C therapy, significantly improving treatment response rates, patient life expectancy and quality of life. Global prevalence estimates show that about 71 million people are infected with HCV representing about 1% of the world's population, with an annual incidence of 1.75 million new infections. Worldwide, approximately 6.2% of human immunodeficiency virus (HIV) infected people are also co‐infected with hepatitis C virus (HCV) (2.28 million), which translates to 2.4% of people living with HIV.

HCV affects over 80% of all intravenous drug users (PWID) and 6.4% of men who have sex with other men (MSM).^1^ Of the approximately 10.2 million people with chronic HCV in Europe, most are living in Eastern Europe (6.7 million) followed by Western Europe (2.3 million) and Central Europe (1.2 million), with an estimated HCV European prevalence of 1.5%.^4^, ^5^, ^6^ Unsafe health care practices and injection drug use perpetuates HCV transmission, in parallel with increasing HCV‐related liver disease burden, as a result of an ageing population infected during peak HCV epidemics in past decades. HCV‐related disease represented the 7th global cause of death in 2015 (WHO data), and one of the main reasons for liver transplantation.

In 2016, the World Health Organization defined targets to eliminate HCV infection as a major public health threat by 2030, aiming at 80% reduction in new infections, 65% reduction in mortality and at least 80% of patients engaged in treatment. Portugal has been identified as one European country with a friendly advocate policy. Since early 2015, SOF and SOF/LDV were the first fully government reimbursed second‐generation DAAs and generalized use of interferon‐free regimens became current practice. After July 2016, the remaining approved DAAs were sequentially added and covered by the same health access policy.

By the end of 2018, according to the national HCV treatment database registry (INFARMED) 20 337 HCV infected patients had started DAA treatment. The global SVR achieved until then was 96.6% among 11 718 patients who had completed treatment and follow‐up evaluation.^7^

The HCV/HIV co‐infected population represents 22.4% (n = 4.957) of those proposed for DAA treatment in Portugal. 85% of these had already initiated HCV therapy. The SVR rate recorded among HCV/HIV co‐infected patients was 95.9% (2.649 patients completed treatment and underwent SVR evaluation).^10^ HCV and HIV co‐infected patients are still considered a priority to engage in HCV treatment due to faster liver disease progression. This paper aimed to describe a cohort of 2133 HCV/HIV co‐infected patients that engaged in HCV treatment with DAA‐containing regimens, from early 2015 until July 2018, reflecting a real‐life clinical experience.

## 2 PATIENTS AND METHODS

The Portuguese Study Group of Hepatitis and HIV Co‐infection (GEPCOI) collected data from 15 hospital centres in Portugal, aiming to better characterize the HCV treatment experience with DAA regimens in HCV/HIV co‐infected populations. A common database was created allowing the participation of hospital centres to input the desired data. Inclusion criteria were adult HCV/HIV co‐infected individuals who started therapy with a DAA interferon‐free regimen, during the period between January 2015 and July 2018. Exclusion criteria were consistent with those defined by national and the European Association for the study of Liver Disease (EASL) guidelines, who do not recommend treatment in patients with limited life expectancy due to non–liver‐related comorbidities, including hepatic carcinoma in palliative care.^2^, ^3^

A multicenter, retrospective, observational study of a real‐life clinical cohort was conducted and included 2133 HCV chronically infected patients, with HIV co‐infection. The study population was medically followed up at 15 different clinical centres across the country (Table 1). Demographic (age, gender and geographic origin), epidemiological (HCV likely route of transmission and date of diagnosis), clinical (hepatic disease stage, presence of cirrhosis), immunological and virological (CD4^+^ lymphocytes count, HCV genotype, HCV plasma RNA quantification and HIV plasma RNA quantification) and treatment (previous response to HCV treatment, DAA regimen, duration and response after treatment) data were collected. Hepatic disease stage was accessed by clinical, laboratory and hepatic ultrasound evaluation, associated with noninvasive methods, such as hepatic elastography and/or APRI and FIB‐4 serum biomarkers, which allowed categorization if cirrhosis was present. Patients with decompensated cirrhosis were not included in this analysis.

**Table 1:** Geographic distribution of participating hospital centres by country region (N = 2133)

Country region	Hospital	Number of patients included
North	Porto^b^	406
	Porto^c^	337
	Gaia/Espinho^f^	127
	Aveiro^l^	39
	Viana do Castelo^n^	20
Centre	Lisbon^a^	332
	Setúbal^d^	211
	Lisbon^e^	185
	Almada^g^	105
	Coimbra^h^	97
	Amadora^i^	93
	Lisbon^m^	35
South	Portimão^j^	89
	Faro^k^	47
Island	Funchal, Madeira^o^	10
Total		2133

^a^ Serviço de Infecciologia e Medicina Tropical, Centro Hospitalar de Lisboa Ocidental, Hospital de Egas Moniz.

^b^ Serviço de Doenças Infecciosas, Centro Hospitalar do Porto.

^c^ Serviço de Doenças Infecciosas, Centro Hospitalar de São João.

^d^ Serviço de Doenças Infecciosas, Centro Hospitalar de Setúbal.

^e^ Serviço de Doenças Infecciosas, Centro Hospitalar Universitário de Lisboa Central, Hospital de Curry Cabral.

^f^ Serviço de Doenças Infecciosas, Centro Hospitalar de Gaia/Espinho.

^g^ Serviço de Infecciologia, Hospital Garcia de Orta, EPE.

^h^ Serviço de Doenças Infecciosas, Centro Hospitalar e Universitário de Coimbra.

^i^ Serviço de Doenças Infecciosas, Hospital Fernando da Fonseca.

^j^ Serviço de Medicina, Centro Hospitalar Universitário do Algarve, Hospital de Portimão.

^k^ Serviço de Doenças Infecciosas, Centro Hospitalar Universitário do Algarve, Hospital de Faro.

^l^ Serviço de Doenças Infecciosas, Centro Hospitalar do Baixo Vouga.

^m^ Serviço de Medicina 1.4, Centro Hospitalar Universitário de Lisboa Central, Hospital de São José.

^n^ Serviço de Doenças Infecciosas, Unidade Local Saúde Alto Minho.

^o^ Serviço de Doenças Infecciosas Hospital dos Marmeleiros.

Data were collected from individual clinical files. The epidemiological and clinical characteristics of patients treated for chronic hepatitis C with DAAs were evaluated and related to SVR. SVR was defined as HCV RNA undetectable at week 12 or 24 after treatment. When HCV RNA was not available (eg, lost to follow‐up, death), patients were considered as not achieving SVR12. For categorical variables, we used the chi‐square test and for continuous variables Student's *t* test for independent samples.

Liver cirrhosis was considered when patients had a fibroscan score (FS) above 12.5 KPa or when having an APRI score above 1.0 and a FIB‐4 score above 3.25.^11^, ^12^ Multivariate logistic regression was used to determine whether there were any correlations between SVR and those independent variables with significant differences (*P* < .05) and of clinical interest. The predictive variables included were defined a priori and included gender, age, HCV genotype, previous HCV treatment, presence of cirrhosis, HCV RNA and DAA regimen used (SOF/RBV vs others). *Odds ratios* (ORs) and *P*‐values are presented where applicable. The significance level established for all analyses was .05. Data were analysed using the statistical software SPSS statistics, version 15.

## 3 RESULTS

A total of 2133 patients were included in this study. Demographic characterization revealed a male predominance of 83% (n = 1769) and an average age of 46 years old (21‐79). As expected, the vast majority (96%) were born in Portugal. The second most common geographic origin was represented by 49 African patients (2.3%), from Guinea Bissau, Angola, Mozambique, Cape Verde and São Tomé and Principe. Intravenous illicit drug use was the most frequent transmission route for HCV acquisition and was present in 91% (n = 1840) of patients, while sexual transmission was assumed in 8% (n = 171) of cases.

Considering previous exposure to HCV treatment, 69% (n = 1458) were naïve and the remaining 31% (n = 666) were past nonresponders mostly to dual therapy, based on pegylated interferon alpha plus RBV (PegIFN + RBV). Only nine patients (0.4%) had been previously exposed, without virologic response, to pegylated interferon alpha plus RBV plus a first generation protease inhibitor, namely telaprevir (TPV), boceprevir (BOC) or simeprevir (SMV).

Genotype prevalence found in this population was as follows: G1 67.7% (n = 1445); G2 1.2% (n = 25); G3 15.3% (n = 326); G4 15.7% (n = 334); and G5/G6 0.1% (n = 3).

The stage of hepatic disease was assessed by clinical, laboratory and ultrasound evaluation. Additionally, noninvasive methods such as elastography were performed in most patients (n = 1972, 92.5%). One hundred and sixty‐one patients (7.5%) did not have hepatic elastography. Therefore, seromarkers for APRI and FIB4 scores were determined, considering as cutoff values, 1.0 and 3.25, respectively. The joint evaluation of elastography and seromarkers made it possible to identify 1697 (79.6%) patients without cirrhosis and 20.4% (n = 436) with compensated cirrhosis (Table 2).

**Table 2:** Study population demographic and HCV baseline characterization

Male gender (%)	83
Mean age (y)	46 (21‐79)
Portuguese origin (%)	96
Intravenous drug use transmission (%)	91
HCV treatment naïve (%)	69
Genotype 1 (%)	67.7
Genotype 3 (%)	15.3
Genotype 4 (%)	15.7
Cirrhosis (%)	20.4
Mean plasma baseline HCV RNA (IU/mL)	4.430.389

Immunological evaluation at baseline, before DAA treatment initiation, revealed a mean lymphocyte CD4^+^ count of 619 cel/mm^3^ and 96% of patients on antiretroviral therapy were virologically suppressed. Besides genotype and clinical evaluation, the prescription of DAA regimens was dictated by national and European therapeutic guidelines and reimbursement national policies. During the study period, the most frequently prescribed regimens are listed in Table 3.

**Table 3:** DAA regimens prescribed (N = 2133)

DAA regimen	Number of patients (%)
SOF/LDV	1784 (83.6)
SOF + RBV	204 (9.6)
SOF + DCV	54 (2.5)
3D	43 (2)
SOF + PR	24 (1.1)
GZV/EBV	15 (0.7)
SOF/VEL	7 (0.3)
SOF + SMV	2 (0.1)

Note

SOF/LDV (sofosbuvir/ledipasvir).

SOF/RBV (sofosbuvir/ribavirin).

SOF + DCV (sofosbuvir plus daclatasvir).

3D (paritaprevir/ombitasvir + dasabuvir).

SOF + PR (sofosbuvir plus pegylated interferon alpha plus ribavirin).

GZR/EBR (grazoprevir/elbasvir).

SOF/VEL (sofosbuvir/velpatasvir).

SOF/SMV (sofosbuvir plus simeprevir).

According to therapeutic guidelines at the time of treatment initiation, almost two thirds of patients were eligible for 12‐week duration regimens (64%, n = 1365), and 34.7% (n = 740) had criteria for 24 weeks of treatment, mostly regarding those infected by genotype 3. Only 19 patients (0.9%) were considered eligible for 8 weeks treatment duration, mostly due to local hospital restriction guidelines at the time. Ribavirin was added in 21.6% (n = 461) of treatment regimens, highlighting the fact that 204 of those patients were infected by genotype 2 or 3 and initially began treatment with SOF plus RBV dual therapy, a regimen recommended by therapeutic guidelines at that time.

**Figure 1:**
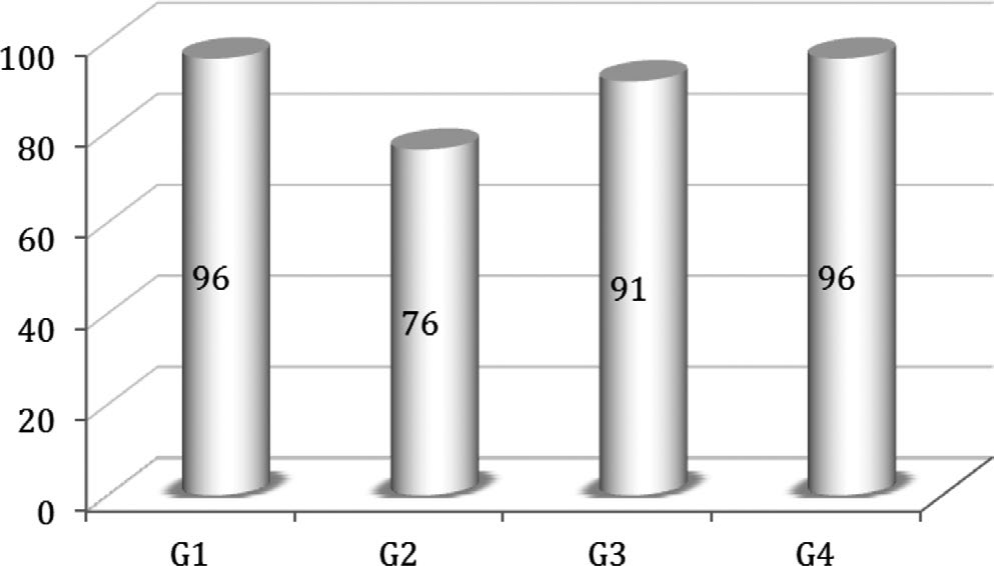
Sustained virologic response rates (%) by HCV genotype (N = 2133)

**Figure 2:**
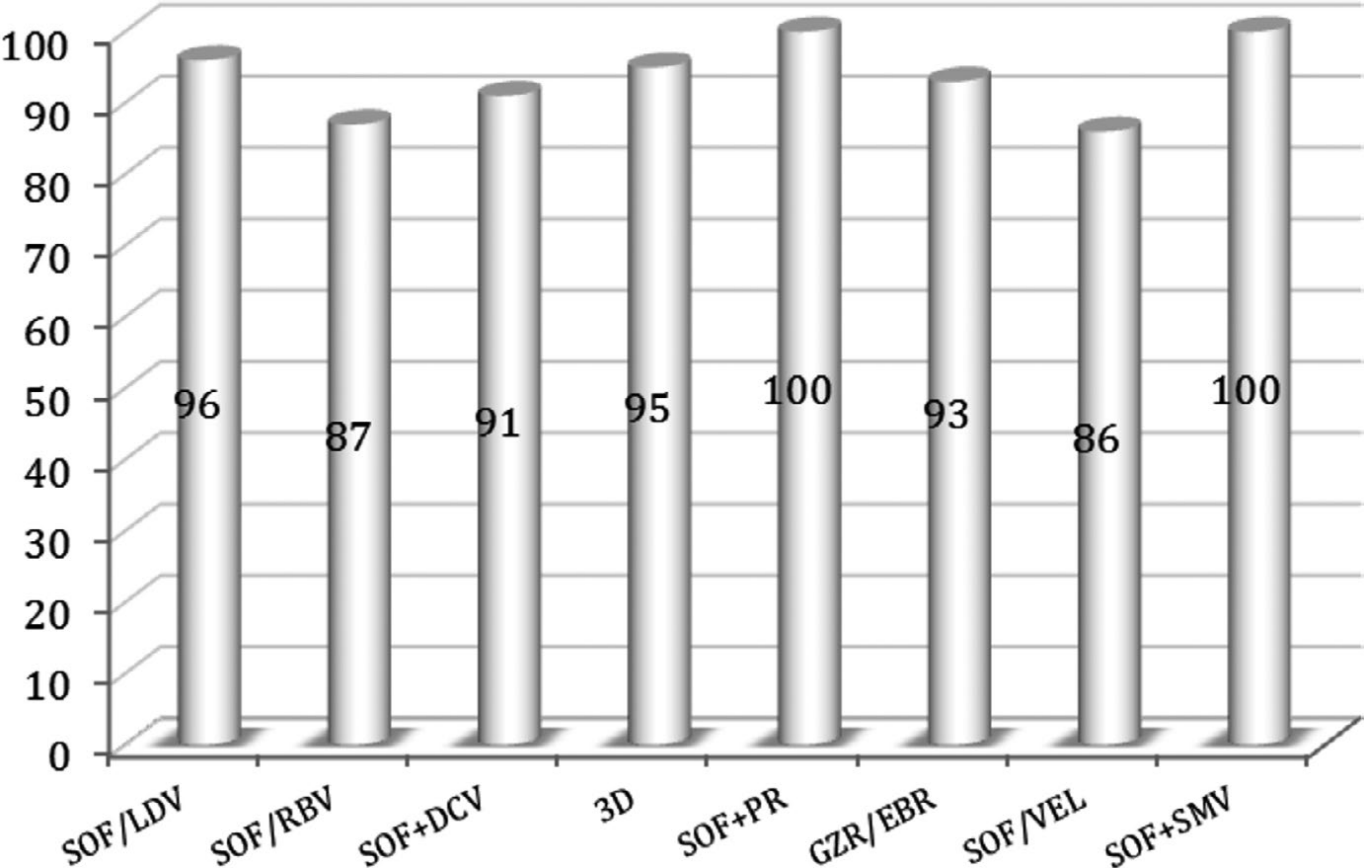
Sustained virologic response rates (%) according to DAA regimen (N = 2133). DAA regimen: number of patients who had sustained virologic response/total of patients who had been treated with the corresponding DAA regimen. SOF/LDV (sofosbuvir/ledipasvir): n = 1712/1784. SOF/RBV (sofosbuvir/ribavirin): n = 178/204. SOF+DCV (fofosbuvir plus daclatasvir): n = 49/54. 3D (paritaprevir/ombitasvir+dasabuvir): n = 41/43. SOF+PR (sofosbuvir plus pegylated interferon alpha plus ribavirin): n = 24/24. GZR/EBR (grazoprevir/elbasvir): n = 14/15. SOF/VEL (sofosbuvir/velpatasvir): n = 6/7. SOF/SMV (sofosbuvir plus simeprevir): n = 2/2

Overall SVR was 95%. The rate of therapeutic failure by relapse (n = 51) or virologic breakthrough (n = 3) was 2.5%. During the study period, 0.9% (n = 19) of patients died and 1.6% (n = 33) were lost to follow‐up. SVR by HCV genotype was as follows: 96% for genotype 1; 76% for genotype 2; 91% for genotype 3; and 96% for genotype 4 (Figure 1). The SVR recorded according to the presence of cirrhosis was 95.4% in those without and 91.7% in those with compensated cirrhosis (*P* < .002). According to the different DAA regimens used, the corresponding SVR rate recorded is represented in Figure 2. Other baseline characteristics did not impact SVR, namely when gender (*P* = .183), patient age (*P* = .68) or HCV RNA level (*P* = .634) were evaluated. Linear regression analysis showed significant differences in treatment response rates when using SOF plus RBV combination (SOF/RBV) in genotype 2 or 3 infected individuals (*P* < .002) and in those with liver cirrhosis (*P* < .02).

## 4 DISCUSSION

The WHO global HCV treatment cascade, published in 2015, documented a current gap of 78% in diagnosis, when considering the 2030 set target of 90%, and a gap of 98% among those engaged in treatment, which translates into 1.76 million people treated until 2015.^8^, ^9^ Across Europe, it is estimated that only 36% of those living with chronic HCV infection have been diagnosed and only about 5% have been treated.^4^ A major barrier to enhancing HCV treatment uptake have been the restrictions established by stakeholders and decision‐makers, including national governments and others, in response to the initially unrealistic high cost of DAA therapies.

To our knowledge, this is one of the largest HCV/HIV co‐infection cohorts ever published regarding treatment with DAAs in a real‐life setting. In an earlier stage, soon after sofosbuvir and other second‐generation DAAs were approval, national treatment recommendations privileged those patients with a more advanced stage of disease. Subsequent HCV national treatment recommendations updates rapidly extended the indication for everyone infected, correlating with our findings, where 69% of our patients were naïve to HCV treatment and 20.4% had advanced liver disease and compensated cirrhosis.^3^

According to the guidelines at that time, 84% of patients were eligible to start treatment with SOF/LDP regimens. The high SVR of 96% registered in this group was concordant with the results published in previous clinical trials.

The second most frequent regimen prescribed was SOF/RBV (10%), recommended at that time for genotype 2 or 3 infected patients, with a significantly lower SVR (87%), related to the use of a, nowadays admitted, less powerful and effective combination. RBV was added in 21.6% of DAA regimens prescribed and over a third of patients (34.7%) completed a period of 24 weeks of treatment. A small percentage of patients (0.9%) completed an 8 weeks short treatment. The lower SVR achieved with the SOF/VEL (86%) regimen should be cautiously interpreted related to the small number of patients so far included (Figure 2). Significantly lower SVR rates were evident in genotype or 3 infected patients (76% and 91%, respectively) and in those with cirrhosis (91.7% vs 95.4% in the absence of cirrhosis).

This study has some limitations related to its retrospective design, as such, the lack of histological confirmation of hepatic cirrhosis, since although it remains the gold standard, liver biopsy has been replaced by noninvasive indirect methods. The extremely rapid evolution of HCV treatment guidelines, recently driven by the approval of pangenotypic DAA regimens, renders the use of some of these therapeutic combinations obsolete, not neglecting, however, the overall high SVR obtained (95%) in this cohort. Current national policy continues to promote the reimbursement of all approved DAAs, allowing for increased and convenient use of pangenotypic regimens.

The HCV/HIV co‐infected population is an obvious target for micro elimination strategy implementation. Micro elimination could be defined as a plan that adapts and allocates available resources and health services, removes barriers and achieves high diagnostic levels and HCV treatment engagement, in this specific population of interest.^8^ These ambitious strategies should be ruled by mathematical modelling estimates, adapted to local epidemiology, in a temporary target setting and depends on a health policy definition, with active involvement of government representatives, technical health and civil society members.^10^

In conclusion, this real‐life Portuguese experience shows a high overall SVR rate of 95%, in a large HCV/HIV co‐infected clinical cohort. The rapid emergence of newer generation DAAs in recent years and the scientific evidence evolution renders the use of some treatment combinations described herein as obsolete.

After a short 3 years use of DAA therapy, a huge clinical experience has been acquired, strengthening the enthusiasm of witnessing the gain in quality of life of our patients. This HCV/HIV co‐infected population is an obvious target for implementation of a micro elimination strategy that could be achieved if the current and, hopefully future trend, in reimbursement of Portuguese health policy will continue. Such a strategy will promote wider access and individualized treatment options, aiming at the elimination of HCV infection in this high‐risk population of people living with HIV.

## CONFLICT OF INTERESTS

AC Miranda has received personal fees from AbbVie, Gilead Sciences, Janssen, Merck Sharp & Dohme, and Roche for lectures, advisory board participation and consultant advise. R Serrão received during 2018 personal fees from Janssen, Merck Sharp & Dohme, Gilead Sciences, Vivv Healthcare for advisory board participation and participation in lectures. M Manata has received personal fees from Gilead Sciences, Janssen, AbbVie, Merck Sharp & Dohme and Roche for lectures, advisory board participation and consultant advice. A Gomes has received personal fees from AbbVie and Vivv Healthcare for advisory board participation and Clinical Trials. C Valente has received personal fees from AbbVie, Gilead Sciences, Janssen, Merck Sharp & Dohme and Vivv Healthcare for lectures and advisory board participation. P Pacheco has received personal fees from Gilead Sciences, Janssen, Merck Sharp & Dohme, AbbVie and Vivv Healthcare for advisory board participation, consultant advice and Clinical Trials. RS Castro received during 2018 fees for participation in lectures or advisory boards for Abbvie, Gilead Sciences, Janssen, Merck Sharp & Dohme and ViiV Healthcare. Remaining authors have no conflict of interest to declare.
